# Consensus‐Building Processes for Implementing Perioperative Care Pathways in Common Elective Surgeries: A Systematic Review

**DOI:** 10.1111/jan.16524

**Published:** 2024-10-09

**Authors:** Lisa Pagano, Oya Gumuskaya, Janet C. Long, Gaston Arnolda, Romika Patel, Rebecca Pagano, Jeffrey Braithwaite, Emilie Francis‐Auton, Andrew Hirschhorn, Mitchell N. Sarkies

**Affiliations:** ^1^ Australian Institute of Health Innovation, Faculty of Medicine, Health and Human Sciences, Macquarie University Sydney New South Wales Australia; ^2^ School of Health Sciences, Faculty of Medicine and Health University of Sydney Sydney New South Wales Australia; ^3^ School of Nursing and Midwifery Western Sydney University Parramatta New South Wales Australia; ^4^ Royal Prince Alfred Hospital, Sydney Local Health District Sydney New South Wales Australia; ^5^ School of Education, Faculty of Education and Arts Australian Catholic University Sydney New South Wales Australia; ^6^ MQ Health, Faculty of Medicine, Health and Human Sciences Macquarie University Sydney New South Wales Australia; ^7^ Implementation Science Academy, Sydney Health Partners University of Sydney Sydney New South Wales Australia

**Keywords:** consensus, implementation science, implementation strategy, perioperative pathways

## Abstract

**Aims:**

To identify and understand the different approaches to local consensus discussions that have been used to implement perioperative pathways for common elective surgeries.

**Design:**

Systematic review.

**Data Sources:**

Five databases (MEDLINE, CINAHL, EMBASE, Web of Science and the Cochrane Library) were searched electronically for literature published between 1 January 2000 and 6 April 2023.

**Methods:**

Two reviewers independently screened studies for inclusion and assessed quality. Data were extracted using a structured extraction tool. A narrative synthesis was undertaken to identify and categorise the core elements of local consensus discussions reported. Data were synthesised into process models for undertaking local consensus discussions.

**Results:**

The initial search returned 1159 articles after duplicates were removed. Following title and abstract screening, 135 articles underwent full‐text review. A total of 63 articles met the inclusion criteria. Reporting of local consensus discussions varied substantially across the included studies. Four elements were consistently reported, which together define a structured process for undertaking local consensus discussions.

**Conclusions:**

Local consensus discussions are a common implementation strategy used to reduce unwarranted clinical variation in surgical care. Several models for undertaking local consensus discussions and their implementation are presented.

**Implications for the Profession and/or Patient Care:**

Advancing our understanding of consensus building processes in perioperative pathway development could be significantly improved by refining reporting standards to include criteria for achieving consensus and assessing implementation fidelity, alongside advocating for a systematic approach to employing consensus discussions in hospitals.

**Impact:**

These findings contribute to recognised gaps in the literature, including how decisions are commonly made in the design and implementation of perioperative pathways, furthering our understanding of the meaning of consensus processes that can be used by clinicians undertaking improvement initiatives.

**Reporting Method:**

This review adheres to the Preferred Reporting Items for Systematic Reviews and Meta‐Analysis (PRISMA) guidelines.

No patient or public contribution.

**Trial Registration:** CRD42023413817

**Summary**

- High levels of clinician autonomy suggest the need for local consensus discussions as a strategy to support implementation into routine practice, however guidance on the practical applications of local consensus discussions is limited.
- We advocate for a systematic approach to using consensus discussions for change in hospitals, such as for care pathway development, which is crucial to advancing collaboration and decision making in health care.

This includes forming dedicated implementation groups, defining standardised pathways, implementing pathways, evaluating success and transparent reporting of key criteria for achieving consensus.

**Abbreviations**
ERAS: enhanced recovery after surgery
MMAT: Mixed Methods Appraisal Tool
PRISMA: Preferred Reporting Items for Systematic Reviews and Meta‐Analysis

## 1 Introduction

Perioperative pathways are designed to standardise the management of surgical patients according to the best available evidence during the preoperative, intraoperative and postoperative periods (Cline et al. 2020; Grocott et al. 2019). The effectiveness of standardised perioperative pathways in reducing unwarranted clinical variation and improving patient outcomes for many elective surgeries is well established (Cline et al. 2020; Rotter et al. 2010; Harrison et al. 2019; Sarkies et al. 2023). However, as more hospitals move towards implementing standardised perioperative pathways into routine practice, there is limited guidance on how best to develop and implement these pathways into different settings. Implementing new interventions or processes into routine practice is complex, requiring a tailored approach to suit the needs of each individual context (Fischer et al. 2016; Jabbour et al. 2018; Pearsall and McLeod 2018; McArthur et al. 2021). For example, it is important to consider the complexities of clinician behaviour change along with system‐level barriers, such as inadequate resources or communication channels, when implementing new interventions (Sarkies, Robinson, et al. 2021; Greenhalgh and Papoutsi 2019).

Numerous strategies exist to improve implementation efforts and integrate interventions of proven efficacy into clinical practice (Powell et al. 2015, 2012). The difficulty lies in selecting an appropriate strategy from a plethora of options available. Implementation strategies can be mismatched to the local contextual circumstances, such as using a clinician education strategy to address an issue of organisational resourcing (Bosch et al. 2007; Kinsman et al. 2009). In many healthcare settings, surgeons and other medical practitioners operate with a high degree of autonomy, often resulting in variability between individual practices (Australian Commission on Safety and Quality in Health Care and Australian Institute of Health and Welfare 2021). This variability is important since many health problems do not have a unique clinical solution and patients have expectations about their care (Sutherland and Levesque 2020). However, given this autonomy, it is important that clinicians are onboard with any proposed changes to standardise care processes. This requires strategies that build consensus among key stakeholders.

‘Conducting local consensus discussions’ is one strategy commonly referred to in the literature to achieve agreement between stakeholders and facilitate implementation (Murphy et al. 1998; Black et al. 1999). Consensus discussions are well established as a method to share information and come to agreement where there are multiple perspectives (Kea and Sun 2015). These discussions may include formal approaches to achieve consensus such as the Delphi (Dalkey and Helmer 1963; Delbecq, Van de Ven, and Gustafson 1975) and Nominal Group Technique, (Van de Ven and Delbecq 1972) or informal or unstructured processes (Kea and Sun 2015; World Health Organization 2014). There is ample guidance available on formal or ‘explicit’ methods to guide consensus discussions, with documented evidence of successful application in clinical practice guideline development (Black et al. 1999; van Zuuren et al. 2022; Tammela 2013). However, the use of local consensus discussions to guide perioperative pathway development or quality improvements within hospitals appears less well documented (Arakawa and Bader 2022; Waltz et al. 2021). Clinical staff often refer to reaching consensus with an inclination towards informal consensus processes or refer to various facets of what may collectively be termed a consensus process (Arakawa and Bader 2022; Banno, Tsujimoto, and Kataoka 2020; Innes and Booher 1999). Often, the steps to build consensus are not clearly documented, such as the level of agreement between participants or whether a leader's suggestion influenced their agreement. Consequently, the optimal approaches to obtain consensus in these situations are unclear.

### 1.1 The Review

Elective hip and knee arthroplasty and spinal surgeries place a significant burden on the healthcare system, (Australian Orthopaedic Association National Joint Replacement Registry (AOANJRR) 2019; Australian Institute of Health and Welfare 2019) with incidences expected to increase in public and private hospital settings (Australian Commission on Safety and Quality in Health Care and Australian Institute of Health and Welfare 2021; Ackerman et al. 2019). While there is evidence of the effectiveness of perioperative pathways in these areas, ongoing unwarranted clinical variation persists (Australian Commission on Safety and Quality in Health Care and Australian Institute of Health and Welfare 2021; Garriga et al. 2019), highlighting the importance of consensus‐based approaches to ensure that the highest standard of care is consistently provided. Understanding how to facilitate consensus discussions among clinicians in healthcare settings is crucial. Firstly, consensus discussions can enhance buy‐in and a sense of ownership of clinical practices, which is important for instigating bottom‐up change. Improved buy‐in can increase the feasibility of implementing changes and can generate momentum for their implementation and sustained adherence over time (Long et al. 2023). Secondly, consensus building supports a focus on patient‐centred care, steering discussions away from siloed or individualistic preferences. By emphasising that patient care is a collective responsibility and that all staff members play a vital role, consensus‐building processes can help to promote a more holistic approach to healthcare delivery (Sarkies et al. 2023) and may assist in reducing unwarranted variation in clinical practice.

The lack of comprehensive guidance on how to devise an implementation plan when employing local consensus discussions necessitates a deeper exploration of its practical applications. Understanding how this strategy has been previously operationalised to implement perioperative pathways is important, since suboptimal application of a strategy may result in poor compliance to the intended pathways (Birken et al. 2017; Powell et al. 2017). Furthermore, there is an imperative to adopt the most efficient approach that will still ensure the strategy's effectiveness, given the time constraints faced by surgeons and other clinicians (Konrad et al. 2010; Yahanda and Mozersky 2020).

### 1.2 Aim(s)

We therefore sought to review the published literature to identify and understand the different approaches to undertaking local consensus discussions that have been used to support the implementation of perioperative pathways for common elective surgeries.

## 2 Methods/Methodology

### 2.1 Design

This systematic review was registered with PROSPERO (registration number CRD42023413817) and has been reported in accordance with the Preferred Reporting Items for Systematic Reviews and Meta‐Analysis (PRISMA) guidelines (see Data S1).

### 2.2 Search Methods

Five databases were searched electronically (MEDLINE, CINAHL, EMBASE, Web of Science and the Cochrane Library) for literature published between 1 January 2000 and 6 April 2023, following consultation with a university librarian. The search was limited to the English language, and terms relevant to the field, population and intervention were combined (Data S2). Electronic database searches were supplemented by snowballing for additional articles from the reference lists of potentially relevant reviews. Reference details for the returned searches were downloaded and imported into the electronic screening program Rayyan where search results were combined, and duplicates removed.

Four authors (LP, OG, JL, GA) independently screened 5% of articles to assess the comprehensiveness of the search strategy, data extraction and interpretation between reviewers. Differing results were discussed and clarified before proceeding with the remaining title and abstract screening. Three authors working in pairs (GA and either LP or OG) independently screened all remaining titles and abstracts. Studies determined to be potentially relevant for inclusion or whose eligibility was uncertain were retrieved and imported into Rayyan for full‐text review. Full‐text articles were each screened independently by two members of the research team (LP, OG, JL, GA, MS) to ascertain eligibility for inclusion. Any discrepancies or disagreements were resolved by consensus.

### 2.3 Inclusion and Exclusion Criteria

The complete inclusion and exclusion criteria are presented in Table 1.

**TABLE 1:** Summary of inclusion and exclusion criteria.

Inclusion criteria	Exclusion criteria
Peer‐reviewed articles Published in English Hospital settings Focus on perioperative pathways for elective surgery of knee, hip or spine Adult populations Used a consensus process or methods to develop or implement the pathway—see matrix below for criteria	Not primary research Full text unavailable (except for conference abstracts) Published before 2000

**Table:** Article includes some level of description of the consensus development and implementation process, which could include one or more of the following:

Category	Example
Starting point for pathway	‐ Developed pathway ‘from scratch’ ‐ Modified already existing pathway, for example, ERAS
Identification of pathway steps to be covered	‐ Literature review and synthesis ‐ Proposed by clinicians/clinician preference ‐ Surveys ‐ Agenda defined by small group and circulated for wider input
Group composition	‐ Multidisciplinary team ‐ Single discipline ‐ Management/administration ‐ Policymakers ‐ Patients/consumers
Additional group information	‐ Details of structure, for example, main group and/or steering committee/chair/facilitator ‐ Number of members ‐ Balance of disciplines (i.e., representation from each discipline)
Process for moving towards consensus	‐ Panel discussions ‐ Face to face meetings ‐ Email ‐ Multidisciplinary meetings
Criteria for consensus	‐ Voting/majority wins ‐ Scoring methods ‐ Nominal group technique/round robin ‐ Delphi/modified Delphi ‐ Description of methods to resolve conflicts
Implementation plan	‘Implementation began with training of all medical, nursing and allied health staff over a 1‐month period with implementation roll‐out staged …’
Measures of success	‐ Implementation fidelity ‐ Clinical outcomes

#### 2.3.1 Type of Study and Publication Type

All peer‐reviewed empirical study designs were considered. This included experimental, observational, descriptive, qualitative and mixed methods study designs. Conference abstracts were considered eligible. Descriptions of non‐empirical studies, expert opinions and articles that were not peer reviewed were excluded.

#### 2.3.2 Population

The population included individuals aged 18 years and older undergoing either: (i) elective total hip or knee arthroplasty or (ii) spinal surgery on any anatomical site. Studies examining paediatric populations were excluded from this review due to the differences in components of pathways making comparison difficult (Manning and Bakel 2021).

#### 2.3.3 Interventions

Included articles examined the development and implementation of a perioperative pathway into hospital settings. To be eligible, studies had to: (i) include sufficient information demonstrating that local consensus discussions were included as part of the decision‐making process and (ii) indicate one or more elements of the consensus process. We defined a consensus process as one where stakeholders engage in discussions that address whether the chosen problem is important and whether the clinical innovation to address it is appropriate, (Powell et al. 2015) with the aim to reach overwhelming agreement. Elements of consensus discussions were determined by review of relevant existing literature and by the research teams' own experiences with using the strategy ‘conducting local consensus discussions’ in implementation efforts. These elements were then incorporated into a matrix table to guide screening decisions (see Table 1). For example, eligible studies may have included some level of description of how the process to achieve consensus was conducted, or the criteria used to determine that consensus was reached.

### 2.4 Search Outcome

The methods and processes used to achieve consensus was the primary outcome of this review.

### 2.5 Data Abstraction

Data extraction focused on several categories: publication details, study characteristics and methodology, settings, participant characteristics, type of surgery, methods of development of pathways via consensus and methods of implementation. Each author involved in data extraction could add additional items to the extraction tool relating to the relevant fields as they arose in the included studies. Core elements of local consensus processes to be extracted were categorised into five sections based on a pre‐determined consensus matrix (Table 1): (i) professions involved in the local consensus discussions; (ii) how tasks or items of the care pathways were defined (e.g., literature reviews vs. local expertise); (iii) group processes to achieve consensus (e.g., face‐to‐face meetings or written methods); (iv) criteria for consensus (e.g., formal methods such as voting); (v) agreed upon methods of operationalising or implementing the pathways. Type of outcome measures utilised by each study relating to measures of adherence to the care pathways and four different clinical outcomes (length of stay, mortality, postoperative complications and hospital readmissions) were extracted. Measures of adherence were considered useful to determine if the study was considering the success of implementation and uptake, rather than just clinical effectiveness.

The data extraction tool was developed by LP and modified by four authors (OG, JL, GA, MS). Prior to full‐text review, a pilot assessment was undertaken by two authors (LP and ReP or OG and RoP) of 10% of included studies, where agreement between authors involved in extraction was assessed. Discrepancies were resolved by discussion or review by another author (MS). Once consistency between authors was achieved, each author (LP, OG, RoP, ReP) extracted data from 25% of the remaining articles independently.

### 2.6 Synthesis

The topics extracted and the methods used in the included studies were synthesised narratively. Extracted elements of local consensus discussions were further aggregated into categories and patterns arising from the data were analysed in Microsoft Excel. Where applicable, descriptive characteristics and frequencies were analysed in SPSS Statistics for Windows, version 29.0. (IBM Corp., Armonk, N.Y., USA). In line with our protocol, meta‐analysis was not attempted due to the heterogeneity between the studies.

### 2.7 Quality Assessment

Two of four authors (LP, OG, RoP, ReP) appraised 10% of included articles and compared results to check consistency. Each author then independently assessed the risk of bias and methodological quality of the included studies using the Mixed Methods Appraisal Tool (MMAT) (Pluye et al. 2009; Hong, Gonzalez‐Reyes, and Pluye 2018). The MMAT was developed for use in reviews that include qualitative, quantitative and multi‐method or mixed methods research. Any discrepancy between authors was resolved by discussion or consultation with a fifth independent reviewer (MS).

## 3 Results/Findings

**FIGURE 1:**
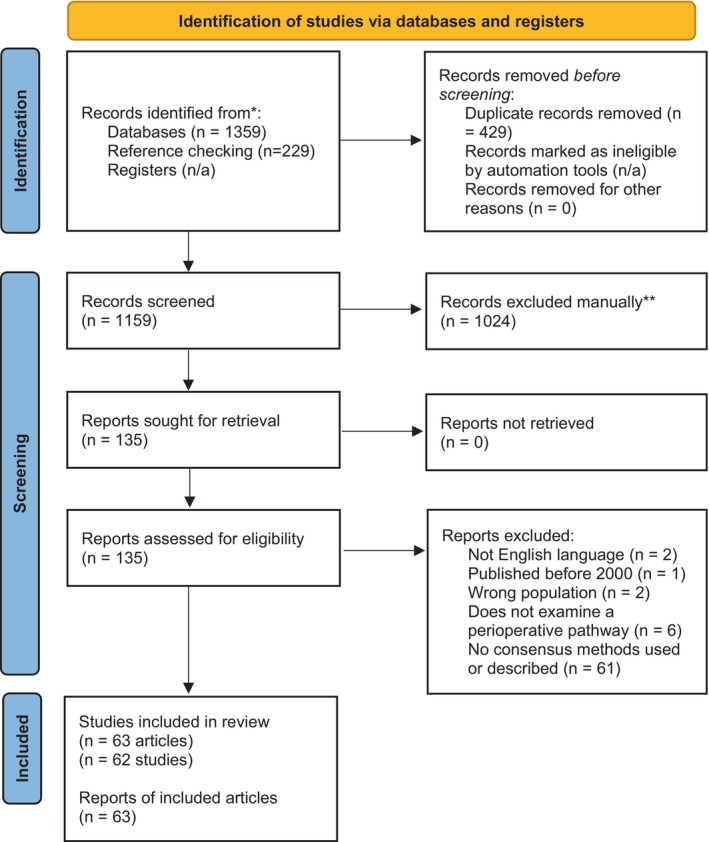
Preferred Reporting Items for Systematic Reviews and Meta‐Analysis (PRISMA**)** flow diagram. Adapted from: Page MJ, McKenzie JE, Bossuyt PM, Boutron I, Hoffmann TC, Mulrow CD, et al. The PRISMA 2020 statement: An updated guideline for reporting systematic reviews. *BMJ* 2021;372:71. doi: 10.1136/bmj.n71

Initial searches yielded 1588 results and 1159 records were available for screening following removal of duplicates. A total of 1024 records were excluded after examination of titles and abstracts, leaving 135 studies for full text screening. A total of 62 studies (*n* = 63 articles; one study reported relevant information in two publications) were considered eligible and included in the review. The screening process and results are displayed in the PRISMA flow chart in Figure 1. The characteristics of included studies are displayed in Table 2.

**TABLE 2:** Characteristics of included studies.

Author, year	Study design	Country	Hospital type	Sites	THA	TKA	Spinal				Pathway type		
							Lumbar	Thoracic	Cervical	NFD	Part or full pathway?	Design principles	New/modified
Alvis et al. (2021)	Non‐concurrent cohort study	USA	VA	One	☑	☑					Part (Anaesthetics)	ERAS	Modified
Angus et al. (2019)	Non‐concurrent cohort study	UK	Tertiary	One						☑	Full	ERAS	Modified
Barber et al. (2017)	Before–after, cohort study	USA	Public	Many	☑						Part (Post‐op)		Modified
Bardiau et al. (2003)	Before–after, cohort study	Belgium	Public	One	☑	☑					Full		New
Blackburn et al. (2016)	Non‐comparative study	UK	Public	One			☑	☑	☑		Full		Modified
Bradywood et al. (2017)	Before–after study	USA	Public	One			☑				Full		New
Chung et al. (2012)	Before–after, cohort study	South Korea	NR	One			☑				Full		New
Cook et al. (2008)	Non‐comparative study	USA	Secondary	One		☑					Full		New
Didden et al. (2019)	Before–after, cohort study	Netherlands	Public, academic	One		☑					Part (Post‐op)		Modified
Eklund, Vodonos, and Ryan‐Barnett (2022)	Controlled, interrupted time series	USA	Tertiary, academic	One	☑	☑					Full		New
El‐Othmani et al. (2021)	Before–after, cohort study	USA	Academic	One	☑	☑					Full	Some ERAS	New
Featherall et al. (2018)	Before–after, controlled cohort study	USA	Private	Many	☑						Full		Modified
Feng et al. (2019)	Before–after, controlled cohort study	China	Public	One			☑				Full	ERAS	Modified
Foni et al. (2020)	Before–after, controlled cohort study	Brazil	Private	One		☑					Full		New
Garg et al. (2021)	Before–after, cohort study	India	Tertiary	One			☑				Full	ERAS	Modified
Gayed et al. (2013)	Before–after, cohort study	USA	VA Hospital	One	☑	☑					Full		New
Ghobrial et al. (2020)	Before–after, controlled cohort study	USA	Academic	One			☑	☑^a^	☑		Full		New
Manning and Bakel (2021)	Case‐matched, controlled cohort study	USA	Academic, tertiary	One	☑						Full	ERAS	Modified
Gwynne‐Jones, Martin, and Crane (2017)	Non‐concurrent cohort study	New Zealand	Public	One	☑	☑					Full	ERAS	Modified
Hall et al. (2019)	Conference abstract	NR	MDC	One						☑	Full	ERAS	Modified
Hawasli et al. (2020)	Non‐comparative study	USA	Tertiary	One			☑				Full	ERAS	Modified
Hebl et al. (2008)	Case‐matched, controlled cohort study	USA	Tertiary	One	☑	☑					Full	ERAS	Modified
Hypnar and Anderson (2001)	Non‐comparative study	USA	Public	One	☑	☑					Full		New
Improta et al. (2015)	Non‐concurrent cohort study	Italy	Tertiary, Academic	One	☑						Full		New
Lampilas et al. (2021)	Before–after, controlled cohort study	France	Public, academic	NR			☑				Full	ERAS	Modified
Larsen, Sørensen, et al. (2008)	Randomised trial	Denmark	Secondary	One	☑	☑^b^					Full		New
Larsen, Hvass, et al. (2008)	Before–after trial	Denmark	Secondary	One	☑	☑					Full		New
Li et al. (2018)	Before–after, controlled cohort study	China	Tertiary	One					☑		Full	ERAS	Modified
Lin et al. (2011)	Non‐randomised trial	Taiwan	NR	One		☑					Full		New
Loftus et al. (2014)	Before–after, controlled cohort study	USA	Acute care	Many		☑					Full		New
MacDonald, Arthur, and Parent (2005)	Non‐comparative study	Canada	Tertiary	One	☑	☑					Full		New
Mertes, Raut, and Khanduja (2013)	Before–after study	UK	NR	One	☑	☑					Full		New
Mudumbai et al. (2016)	Before–after, cohort study	USA	Tertiary, VA	One		☑					Part (Intraop)		Modified
Parkes et al. (2021)	Non‐concurrent before–after, cohort study	UK	Public	One	☑	☑					Full	ERAS	New
Pearson, Moraw, and Maddern (2000)	Before–after, cohort study	Australia	Public	One		☑					Full		New
Pennington, Jones, and McIntyre (2003)	Before–after, cohort study	New Zealand	Public	One		☑					Full		New
Peters, Shirley, and Erickson (2006)	Before–after, cohort study	USA	Tertiary	One	☑	☑					Part (Anaesthetics)		Modified
Raphael, Jaeger, and van Vlymen (2011)	Before–after, controlled cohort study	Canada	Tertiary	One	☑	☑					Full		Modified
Riepen et al. (2021)	Before–after, cohort study	USA	Tertiary	One		☑					Full		New
Scanlon and Richards (2004)	Non‐comparative study	USA	HMO	One			☑				Full		New
Schubert et al. (2021)	Non‐concurrent cohort, interrupted time series	USA	Academic, tertiary	One	☑						Full		Modified
Scott et al. (2013)	Audit	Scotland	Tertiary	Many	☑	☑					Full	ERAS	Modified
Shao et al. (2022)	Before–after, controlled cohort study	China	Tertiary	One			☑				Full	ERAS	Modified
Shaw and Pilot (2016)	Conference abstract	Netherlands	NR	Many	☑	☑					Part (Postop)	‘Rapid recovery’	Modified
Shields et al. (2017)	Before–after study	USA	Tertiary	One			☑				ND		Modified
Sivaganesan et al. (2019)	Before–after, cohort study	USA	Tertiary	One			☑		☑		Full		New
Smith et al. (2019)	Before–after, controlled cohort study	USA	Tertiary	One			☑				Full	ERAS	Modified
Soffin et al. (2020)	Randomised trial	USA	Tertiary	One			☑				Full	ERAS	New
Soffin, Wetmore, et al. 2019	Before–after, cohort study	USA	NR	One					☑		Full	ERAS	Modified
Soffin, Vaishnav, et al. (2019)	Before–after, cohort study	USA	NR	One			☑				Full	ERAS	Modified
Stowers et al. (2016)	Non‐concurrent cohort study	New Zealand	Public	One	☑	☑					Full	ERAS	Modified
Stratton (2000)	Before–after study	USA	Private	One	☑	☑					Full		Modified
van der Sluis et al. (2015)	Before–after, cohort study	Netherlands	Public	One		☑					Full		Modified
Vanhaecht et al. (2005)	Interrupted time series	Belgium	Tertiary, academic	One		☑					Full		New
Walker et al. (2020)	Before–after, cohort study	USA	Public	One			☑				Full		New
Wallny et al. (2014)	Descriptive	Germany	Public	One		☑					Full		Modified
Walter et al. (2007)	Before–after, cohort study	USA	Community	One	☑	☑					Full		Modified
Wang et al. (2020)	Case‐matched, controlled cohort study	China	Tertiary	One			☑				Full	ERAS	Modified
Wang et al. (2022)	Before–after, cohort study	China	Tertiary	One			☑				Full	ERAS	Modified
Wang et al. (2022)	Case‐matched, controlled cohort study	China	Tertiary	One			☑				Full	ERAS	Modified
Woo et al. (2000)	Cohort study	Canada	Public	One	☑	☑					Part (Preop)		New
Yang et al. (2016)	Randomised trial	China	Tertiary	One	☑						Full		Modified
Young et al. (2021)	Non‐concurrent cohort study	USA	Academic community	One			☑	☑	☑		Full	ERAS	Modified

Abbreviations: ERAS, Enhanced Recovery After Surgery; HMO, health maintenance organisation; MDC, multi‐disciplinary centre; NFD, not full described; NR, not recorded; THA, total hip arthroplasty; TKA, total knee arthroplasty; UK, United Kingdom; USA, United States of America; VA, veterans association.

^a^ Restricted to thoracolumbar.

^b^ Also includes unicompartmental knee arthroplasty.

### 3.1 Study Design

All included studies used quantitative methods. Majority of studies were cohort studies (Table 2). Two peer‐reviewed conference abstracts were also included.

### 3.2 Study Quality Appraisal

All articles were appraised using the MMAT (Data S3). The quality of reporting was variable between studies. Included randomised controlled trials demonstrated low risk of bias. For non‐randomised studies, methodological limitations were common and existed primarily due to lack of accounting for confounders in the design and analysis, or incomplete reporting of outcome data. Within some descriptive studies, there was high risk of non‐response bias or poor reporting of statistical analyses.

### 3.3 Study Characteristics

Most studies were conducted in the United States of America (*n* = 28, 45%) or Europe (*n* = 13, 21%). Hospital settings were widely variable with studies occurring in public, private, tertiary, academic and veterans' association hospital contexts. Most studies examined implementation in one site only (*n* = 55, 89%). Development and implementation of a perioperative pathway was evaluated for total knee arthroplasty (*n* = 12, 19%), total hip arthroplasty (*n* = 6, 10%), both hip or knee arthroplasty (*n* = 20, 32%) or elective spinal surgery (*n* = 24, 39%). Of the studies that developed a pathway for elective spinal surgery, spinal fusion was the most common procedure. Over half of the included studies (*n* = 35, 56%) modified an existing perioperative pathway and the remaining 27 studies (44%) developed a new pathway. Enhanced recovery after surgery (ERAS) principles (ERAS Society 2024) were utilised or formed the basis of perioperative pathways in 23 (37%) studies.

### 3.4 Components of the Consensus Processes Utilised

**FIGURE 2:**
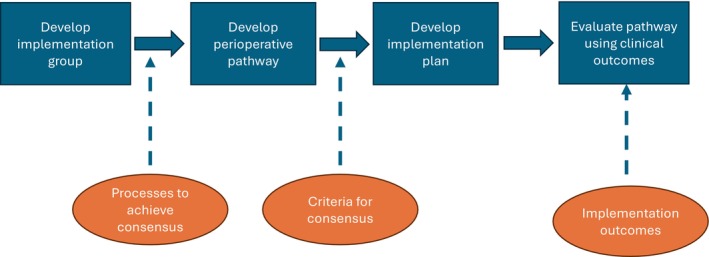
Items representing the process for conducting local consensus discussions to develop and/or implement perioperative pathways derived from the included studies. Items adapted from the pre‐determined consensus matrix developed by authors. Blue boxes represent the items consistently reported across included studies. Orange circles indicate the items not consistently reported and those which are recommended to be included in future studies for clarity of reporting.

Reporting of local consensus discussion processes varied substantially across the included studies, however, four elements from the consensus matrix were consistently reported (Figure 2). These elements related to the professions involved in developing the pathways and how pathways were developed, operationalised and evaluated.

**FIGURE 3:**
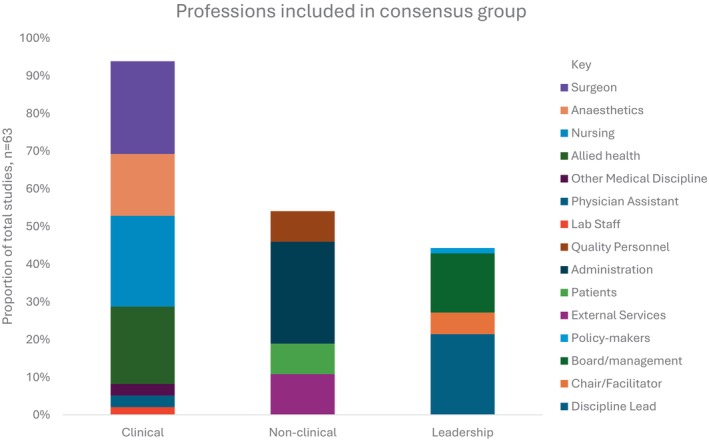
Graphical representation of the proportion of different professions involved in the consensus groups. Professions are divided into three subgroups: clinical disciplines, non‐clinical and leadership professions.

The constituent disciplines of each consensus group were described by most studies (*n* = 61, 98%) and are displayed in Figure 3. Professions involved in the consensus process included clinical disciplines, non‐clinical professions and/or leadership. Surgeons (*n* = 47, 76%) and nursing (*n* = 46, 74%) were the principal clinical disciplines represented. The combination of surgeons, nursing, anaesthetics and allied health was the most prevalent grouping of clinical disciplines (*n* = 17, 27%), followed by surgeons, nursing and allied health (*n* = 14, 23%). Ten studies (16%) referred to the inclusion of a multidisciplinary team without any further explanation of disciplines involved. Non‐clinical professions such as hospital administration and patients were mentioned in 28 (45%) studies, while leadership or management were mentioned in 24 (39%) studies. When comparing studies that modified existing pathways to those that developed new pathways, the involvement of anaesthetists (31% vs. 61%) and external services (22% vs. 0%) were more commonly reported.

**FIGURE 4:**
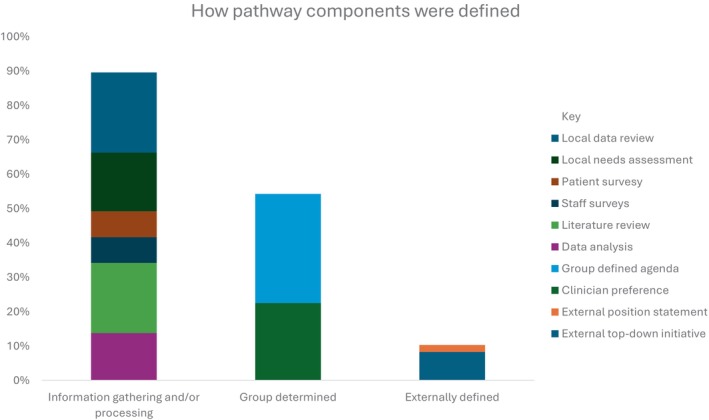
Graphical representation of the different methods reported by studies to determine the individual components of the perioperative pathways and the proportion of studies that used each method. Methods are grouped into three categories: information gathering and/or processing, group determined and externally defined.

Almost all studies (94%) articulated how components of the perioperative pathways were determined; however, there was significant heterogeneity in the processes used to determine these core components (Figure 4). Pathway components were defined either solely or in combination with other methods. Information gathering and processing methods were commonly utilised (*n* = 55, 89%). Over half of studies (56%) reported reviewing local data to clarify the needs of their context and 31 (50%) studies examined literature or conducted a literature review to determine evidence‐based components of care that should make up the pathways. Using group or individual preferences were also described (*n* = 32, 52%). For example, some studies stated that the group members defined the components of the pathways. A small number of studies indicated that they used external or existing position statements (*n* = 7, 11%).

**FIGURE 5:**
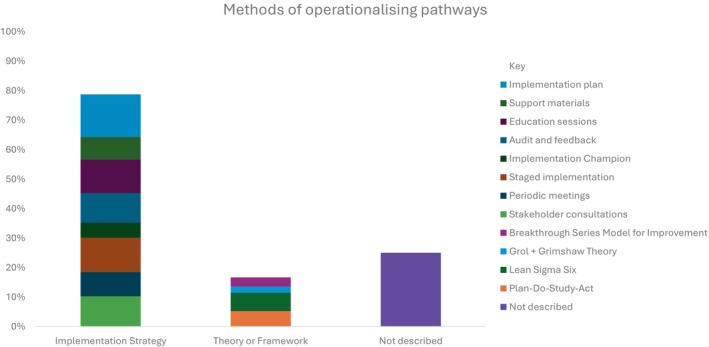
Graphical representation of the different methods reported by studies to operationalise the perioperative pathways and the proportion of studies that used each method. Methods were aggregated into two subgroups: implementation strategy focused or driven by a theory or framework.

Most studies (*n* = 46, 74%) provided some description of the implementation strategies or methods used to operationalise the pathways (Figure 5). Many studies simply reported the use of implementation strategies to guide implementation however, 10 (16%) studies reported on an implementation science theory or framework underpinning the implementation phase.

Perioperative pathways were evaluated using measures of adherence/fidelity and clinical outcome measures (see Table S1). Most studies (*n* = 59, 95%) measured effectiveness through clinical outcomes such as length of stay, while 24 (39%) studies included a measure of adherence to the implemented pathways in their evaluation (see Table S1). Twenty‐two (35%) studies evaluated pathways through both clinical and adherence outcome measures. Only one study did not report any outcome measure.

There was little difference in the components used to develop and implement pathways, regardless of whether studies had developed a new pathway or modified an existing pathway. However, studies that modified existing pathways more frequently reported using stakeholder consultations (44% modified pathways vs. 22% new pathways) or educational resources (39% modified pathways vs. 11% new pathways) to guide implementation, and clinician preferences to establish components of pathways (33% modified pathways vs. 22% new pathways). Conversely, more studies that developed new pathways reported using implementation champions (26% new pathways vs. 11% modified pathways).

Items that were not adequately reported were group processes to conduct consensus decisions (not reported = 49, 79%) and the criteria for determining consensus (not reported = 50, 81%). Further, nearly all studies (*n* = 57, 92%) did not report on a description of methods to resolve conflicts between participants involved in the consensus process. These items have been added to Figure 2 to represent how it would be beneficial for future studies to include these items in reporting.

### 3.5 Emergent Consensus Process Models

A structured process for using local consensus discussions to develop and implement a perioperative pathway within a clinical context was identified, delineated by four pivotal steps (Figure 2). The steps were determined based on the reporting frequency of aggregated variables and included: (i) establish an ‘implementation’ group to develop and implement pathways using a consensus approach; (ii) determine the core components to be included in the pathways using consensus methods; (iii) operationalise or implement the perioperative pathways; (iv) evaluate the success of the perioperative pathway itself and/or the implementation of the pathway.

Table 3 portrays the 29 different process models identified from the included studies to develop and implement care pathways based on these four steps. Only studies that contained an adequate description for each step were used to construct the process models (*n* = 46, 74%). The most common reasons for articles being excluded from this analysis was insufficient reporting of how pathways were operationalised (*n* = 12), how items within the pathways were defined (*n* = 1) or both (*n* = 4). There was substantial variability in the combinations utilised to attain consensus. The most prevalent methods involved having only clinicians in consensus groups, use of information gathering methods or clinician preferences to define tasks and a reliance on strategy‐focused implementation plans to operationalise pathways rather than theory informed. The 29 models comprised six options for group formation (e.g., A: clinical vs. B: clinical + non‐clinical), six options for development of items within the pathways (e.g., A: information review vs. B: literature + group experience), two options for operationalisation (A: implementation strategy vs. B: implementation strategy + theory) and two options for evaluation (A: clinical outcomes vs. B: clinical outcomes + compliance).

**TABLE 3:** Resultant process models of ‘conducting local consensus discussions’ utilised by the included studies.

Model	Groups involved			How components identified			Operationalise		Evaluate		No. (%)
	Clinical	Non‐clinical	Leadership	Information review	Group decided	External	Strategy based	Theory	Compliance	Clinical outcomes	
1	☑			☑			☑		☑	☑	4 (6%) (Alvis et al. 2021; Feng et al. 2019; Sivaganesan et al. 2019; Wang et al. 2020)
2	☑			☑			☑			☑	4 (6%) (Manning and Bakel 2021; Foni et al. 2020; Larsen, Sørensen, et al. 2008; Walker et al. 2020)
3	☑			☑			☑		☑		1 (2%) (Bardiau et al. 2003)
4	☑			☑			☑	☑	☑	☑	1 (2%) (Blackburn et al. 2016)
5	☑			☑	☑		☑			☑	5 (8%) (Angus et al. 2019; Featherall et al. (2018); Garg et al. 2021; Hawasli et al. 2020; MacDonald, Arthur, and Parent (2005)
6	☑			☑	☑		☑		☑	☑	1 (2%) (Li et al. 2018)
7	☑			☑	☑		☑	☑		☑	1 (2%) (Gayed et al. 2013)
8	☑			☑	☑	☑	☑			☑	1 (2%) (Gwynne‐Jones, Martin, and Crane 2017)
9	☑	☑		☑			☑			☑	2 (3%) (Chung et al. 2012; Wang et al. 2022)
10	☑	☑		☑			☑		☑	☑	1 (2%) (Smith et al. 2019)
11	☑	☑		☑	☑		☑			☑	4 (6%) (Ghobrial et al. 2020; Shaw and Pilot 2016; Walter et al. 2007; Yang et al. 2016)
12	☑	☑		☑	☑		☑	☑		☑	1 (2%) (Didden et al. 2019)
13	☑	☑		☑	☑		☑		☑	☑	1 (2%) (Soffin Wetmore, et al. 2019)
14	☑	☑		☑			☑	☑	☑	☑	1 (2%) (Stowers et al. 2016)
15	☑	☑		☑			☑	☑		☑	2 (3%) (El‐Othmani et al. 2021; Schubert et al. 2021)
16	☑	☑	☑	☑			☑		☑	☑	1 (2%) (Lampilas et al. 2021)
17	☑	☑	☑	☑	☑		☑	☑	☑	☑	2 (3%) (Bradywood et al. 2017; Parkes et al. 2021)
18	☑	☑	☑	☑	☑		☑			☑	2 (3%) (Hypnar and Anderson 2001; Mudumbai et al. 2016)
19	☑	☑	☑	☑	☑		☑		☑	☑	1 (2%) (Young et al. 2021)
20	☑	☑	☑		☑		☑		☑		1 (2%) (Eklund, Vodonos, and Ryan‐Barnett 2022)
21	☑	☑	☑	☑	☑	☑	☑			☑	1 (2%) (Improta et al. 2015)
22	☑	☑	☑			☑	☑			☑	1 (2%) (Vanhaecht et al. 2005)
23	☑	☑	☑	☑			☑	☑		☑	1 (2%) (Larsen, Hvass, et al. 2008)
24	☑		☑	☑	☑	☑	☑			☑	1 (2%) (Scanlon and Richards 2004)
25	☑		☑	☑	☑		☑	☑		☑	1 (2%) (van der Sluis et al. 2015)
26	☑		☑	☑	☑		☑			☑	1 (2%) (Stratton 2000)
27	☑		☑	☑			☑		☑	☑	1 (2%) (Soffin, Vaishnav, et al. 2019)
28			☑	☑	☑		☑		☑	☑	1 (2%) (Soffin et al. 2020)
29		☑	☑	☑		☑	☑		☑	☑	1 (2%) (Scott et al. 2013)

As an example, Gulotta et al. (2011) conducted local consensus‐based discussions to develop and implement a ‘fast track’ protocol for patients undergoing total hip replacement. To achieve this aim, the consensus group comprised solely clinical disciplines including surgeons, anaesthetics, nursing and allied health. Included components of the pathway were reportedly determined by information gathering and processing methods in the form of a literature review. Pathways were operationalised using implementation strategies including the use of implementation champions, education sessions and educational handouts. Clinical and/or health service outcome measures were analysed to evaluate the success of the new protocol.

## 4 Discussion

We examined the processes employed by healthcare organisations when conducting local consensus discussions to develop and implement perioperative pathways for common elective surgeries. Our findings contribute to the limited existing knowledge of how this implementation strategy has been used previously, by synthesising the data into four key steps across the included studies. Using these four categories we classified studies with sufficient data as following one of 29 emergent process models (Table 3). These findings make explicit the decisions that are commonly made in the design and implementation of perioperative pathways and provide guidance for those seeking to implement consensus processes.

### 4.1 Variation in Reporting—Consensus Discussions

A key finding was the substantial variation in reporting of how local consensus discussions took place. Professions involved in the consensus groups and how pathway components were defined were consistently reported items, whereas how groups reached agreement on what should be included within each pathway was scarcely reported. This may be as simple as stating that discussions continued until all members of the group agreed with the pathway components, regardless of whether some had reservations. Few studies included a description of how conflicts were resolved when agreement could not be reached, which is important as it is likely to be a barrier to implementation in many settings (Elliott 1999). This pattern echoes previous literature examining the reporting quality of consensus methodology in biomedicine and clinical practice, where group composition is often well described yet evidence indicates that reporting of consensus methods overall could be improved (van Zuuren et al. 2022; Moher et al. 2010). Of note, the number of consensus panel members, definition of consensus used and the agreed consensus thresholds have been inconsistently reported throughout other literature on consensus, such as clinical guideline development (van Zuuren et al. 2022; Arakawa and Bader 2022). Failing to report these methods limits our ability to understand how to effectively carry out consensus processes which could impact on adherence to pathways if not all clinicians agree on what is being implemented.

### 4.2 Variation in Reporting—Implementation and Evaluation

Another notable gap remains in studies reporting on the effectiveness of overall implementation. Implementation effectiveness can be examined through adherence to various components of the perioperative pathways, as well as downstream impacts on health outcomes (Proctor et al. 2011). However, the focus of most included articles was to examine the effects of the pathways on clinical outcomes without reporting on adherence during implementation. This highlights a common disconnect between studies evaluating the effect of an intervention and translating evidence for that intervention into practice (Grimshaw et al. 2012). Understanding implementation adherence allows for corrective action to be taken and helps to uncover whether the pathways themselves were the mechanism driving improved clinical outcomes.

Further, only a small number of studies reported a theory‐driven approach to pathway development, with most studies providing no explanation of how their implementation plan was derived. Combining theory with clinician experience‐based intuition to guide implementation can elicit key barriers to an intervention and inform implementation planning (Powell et al. 2017; Taylor et al. 2023). Reporting theoretical underpinnings to implementation research may also help us to understand where researchers thought change would occur (Lewis et al. 2020). Many studies have reported testing social psychological theories, such as social cognitive theory, to drive their implementation plans, indicating that change was aimed at the individual level (Lewis et al. 2020). In comparison, theories or frameworks used by studies in this review related more to changing processes, such as through Plan–Do–Study–Act cycles, rather than individual behaviour change.

### 4.3 Implementation Determinants—Group Composition

The procedural steps reported within each study to develop consensus‐based pathways may reflect the key determinants that shape successful implementation. Notably, the composition of each ‘consensus’ group was the most frequently reported item; however, the rationale for why different disciplines were included was rarely reported. It is therefore difficult to deduce the exact reasoning for why some professions were or were not included as part of the ‘consensus’ group. The fact that clinicians, especially surgeons and nursing, were commonly involved in the ‘consensus’ group is unsurprising, considering that these clinicians are likely to have expert knowledge of pathway components, as well as hospital processes that would need to be considered in the decision‐making process. Since consensus can be thought of as a type of ‘co‐production’, involving those who are intended to adopt the pathways is likely to accelerate the process of implementation and improve patient outcomes (Bain and Hansen 2020; Rycroft‐Malone et al. 2013). This may be crucial to achieve buy‐in in settings where there are competing clinical priorities for staff on the ground, and where suboptimal adoption may result from low levels of clinician awareness or when the health conditions of focus are deemed a lower priority (Jabbour et al. 2018). The impact of panel size on efficiency of the consensus process also warrants consideration. Determining the balance between accommodating for more perspectives within a larger team versus achieving quicker consensus with a smaller, more focused group should be considered.

Interestingly, the role of leadership did not feature prominently in the included studies, despite healthcare implementation trials often emphasising the role of engaging leadership to drive innovations and boost clinicians' beliefs in the feasibility of change (Sarkies et al. 2021; White et al. 2021; Brainard and Hunter 2015; Weiner 2009; Francis‐Auton et al. 2023). It is possible that the engagement of surgeons and clinical staff most likely to be impacted by pathway modification were prioritised. This may reflect the view that behaviour change is needed at the team level rather than the systems level (Weiner 2009). Alternatively, leadership may have been involved but was underreported in the included studies.

### 4.4 Formal Versus Informal Processes

Few studies in this review utilised common formal consensus methods. Formal methods such as the Delphi can be time consuming and costly to administer and may not always be feasible or necessary in healthcare contexts with competing time pressures (Kea and Sun 2015; de Meyrick 2003). Healthcare agencies may therefore choose to move towards more informal methods to reach agreement, where clinicians are leading change (de Meyrick 2003). Alternatively, formal processes may be considered most relevant the first time something is done in a larger setting but are not required when one is locally engaging in a process that has been successfully implemented elsewhere.

### 4.5 Recommendations

1. Clinicians and researchers should carefully plan their use of consensus discussions by following the four key steps identified and considering the additional factors outlined in Figure 2, for example, determining the criteria for when consensus is reached.
2. Increased transparency of the methods used to develop perioperative pathways and how studies have approached informal consensus discussions can be achieved by clear and consistent reporting of all four aspects listed in the process model (Figure 2 and Table 3).
3. Future research should examine different formal and informal consensus processes to compare when they are required and their impact on overall implementation. A better understanding of which consensus approaches work more effectively to implement different interventions within different healthcare settings can help clinicians and researchers to know what would work in their organisation and guide implementation planning. This calls for the development of research questions that focus on understanding how consensus processes function as part of implementation strategies.
4. It is important to add in measures of fidelity to the newly implemented interventions to examine effectiveness and allow for prompt course‐corrective action.

### 4.6 Limitations

This review employed a systematic search across five large academic databases. However, grey literature or unpublished quality improvement projects were not included, which may contain more detail on local consensus discussion processes than studies published in academic journals. This may mean that certain studies examining perioperative pathway development using the strategy ‘conducting local consensus discussions’ may have been missed. Despite these limitations, we believe our comprehensive search strategy retrieved a relatively complete identification of published peer‐reviewed studies in the field when compared to other reviews examining similar fields (Rotter et al. 2010; Heymans et al. 2022). Multiple group meetings were conducted to ensure consistent coding of themes. Nevertheless, the limited information available in the reported study methods and inconsistent description of consensus discussions meant that accurately detecting consensus processes was time‐consuming. Only articles where reported information was clearly stated were coded; articles that implied but did not explicitly identify a particular method were coded as ‘not reported’ and did not contribute to our emergent models.

## 5 Conclusions

This review synthesises the evidence of how local consensus discussions have been applied to develop and implement perioperative pathways. We found 29 different variations of applications of consensus discussions in past research when defined in four key categories. Reporting of key criteria to achieve consensus is necessary to further our understanding of optimal approaches to conduct local consensus discussions to implement perioperative pathways. The factors identified in our study may provide useful guidance on how healthcare organisations may adapt similar consensus processes in the design and implementation of perioperative pathways.

## Author Contributions

Lisa Pagano, Janet C. Long, Gaston Arnolda, Jeffrey Braithwaite and Mitchell N. Sarkies were responsible for the overall design of this review. Lisa Pagano conducted the initial database search, with additional input from Oya Gumuskaya, Janet C. Long and Mitchell N. Sarkies. Article screening was conducted by Lisa Pagano, Oya Gumuskaya, Janet C. Long, Gaston Arnolda and Mitchell N. Sarkies. Data extraction and risk of bias assessment was conducted by Lisa Pagano, Oya Gumuskaya, Romika Patel and Rebecca Pagano, under the supervision of Mitchell N. Sarkies. Data analysis and synthesis were conducted by Lisa Pagano with additional input from Janet C. Long and Mitchell N. Sarkies. The manuscript version was drafted by Lisa Pagano. Lisa Pagano, Oya Gumuskaya, Janet C. Long, Gaston Arnolda, Rebecca Pagano, Emilie Francis‐Auton, Andrew Hirschhorn, Jeffrey Braithwaite and Mitchell N. Sarkies contributed to the final versions of the manuscript. All authors read and approved the final manuscript.

## Ethics Statement

The authors have nothing to report.

## Consent

The authors have nothing to report.

## Conflicts of Interest

The authors declare no conflicts of interest.

## Peer Review

The peer review history for this article is available at https://www.webofscience.com/api/gateway/wos/peer‐review/10.1111/jan.16524.

## Supporting information


**Data S1.**: Preferred Reporting Items for Systematic Reviews and Meta‐Analysis (PRISMA) checklist. Completed reporting guideline that this article adheres to according to the PRISMA standards.

**Data S2.**: Example of search strategies for academic databases used in this review.

**Data S3.**: Quality appraisal of included articles using Mixed Methods Appraisal Tool (MMAT).

**Data S4.**: Count of studies that utilised single method or category only.

## Data Availability

All data relevant to the study are included in the article or uploaded as supplemental information.
