# Supplementary material for: Consensus‐Building Processes for Implementing Perioperative Care Pathways in Common Elective Surgeries: A Systematic Review
Source: J Adv Nurs. 2024 Oct 9;81(11):7311–31. doi: 10.1111/jan.16524 (PMC12535325; doi:10.1111/jan.16524)
Supplement: Supplementary file 2 — Data S2.: Example of search strategies for academic databases used in this review. [file JAN-81-7311-s004.docx]

**ADDITIONAL FILE 2: EXAMPLES OF SEARCH STRATEGIES**

**Example search strategy for OVID databases**

| # | Query (Medline) | Number of results returned |
| --- | --- | --- |
| 1 | Critical Pathways/ | 7707 |
| 2 | ((clinical or critical or perioperative or surgical or integrated care or care or enhanced recovery or controlled or rapid recovery) adj3 (path or pathway*)).ti,ab. | 30928 |
| 3 | or/1-2 | 34926 |
| 4 | arthroplasty, replacement, hip/ or arthroplasty, replacement, knee/ | 57756 |
| 5 | 3 and 4 | 351 |
| 6 | Spinal Fusion/ | 30912 |
| 7 | diskectomy/ or laminectomy/ or laminoplasty/ | 16467 |
| 8 | microdiscectomy.mp. | 1092 |
| 9 | laminotomy.mp. | 866 |
| 10 | spin$ surg*.tw. | 19158 |
| 11 | *cervical vertebrae/su [Surgery] | 7517 |
| 12 | *lumbar vertebrae/su [Surgery] | 11809 |
| 13 | *thoracic vertebrae/su [Surgery] | 3765 |
| 14 | 6 or 7 or 8 or 9 or 10 or 11 or 12 or 13 | 64129 |
| 15 | 3 and 14 | 143 |
| 16 | 5 or 15 | 493 |

**Example search strategy for EBSCOhost**

| # | Query (CINAHL Complete) | Number of results returned |
| --- | --- | --- |
| 1 | (MH "Critical Path") OR "critical pathways [mesh terms]") OR ( (MH "Health Care Delivery, Integrated") OR (MH "Enhanced Recovery After Surgery") OR "clinical pathway" OR "care pathway" OR "perioperative path*" | 23168 |
| 2 | ""spine surgery"" OR (MH "Thoracic Surgery+") | 67432 |
| 3 | (MH "Spinal Fusion") OR "spinal fusion" | 12632 |
| 4 | (MH "Diskectomy") OR "discectomy" | 3956 |
| 5 | (MH "Laminectomy") OR "laminectomy" OR (MH "Laminoplasty") | 3542 |
| 6 | “microdiscectomy” | 339 |
| 7 | S2 OR S3 OR S4 OR S5 OR S6 OR S7 | 84165 |
| 8 | S1 AND S8 | 280 |
| 9 | (MH "Arthroplasty, Replacement, Knee+") OR (MH "Arthroplasty, Knee, Unicompartmental") OR (MH "Arthroplasty, Replacement, Hip") | 37021 |
| 10 | S1 AND S10 | 203 |
| 11 | S11 OR S9 Limit published date 2000 01 01 | 386 |

**Example search strategy for Cochrane**

ID Search Hits

#1 MeSH descriptor: [Critical Pathways] explode all trees 273

#2 MeSH descriptor: [Arthroplasty, Replacement, Knee] explode all trees 3461

#3 MeSH descriptor: [Arthroplasty, Replacement, Hip] explode all trees 2425

#4 (#2 OR #3) 5489

#5 (#1 AND #4) 14

#6 (spinal surgery):ti,ab,kw 14214

#7 (spine surgery):ti,ab,kw 6324

#8 (spin* surg*):ti,ab,kw 20696

#9 (laminotomy):ti,ab,kw 81

#10 (spinal fusion):ti,ab,kw 2914

#11 (laminectomy):ti,ab,kw 889

#12 (microdiscectomy):ti,ab,kw 284

#13 (diskectomy OR discectomy):ti,ab,kw 1828

#14 (laminoplasty):ti,ab,kw 157

#15 (#6 OR #7 OR #8 OR #9 OR #10 OR #11 OR #12 OR #13 OR #14) 22072
#16 (#1 AND #15) 2
#17 (#5 OR #16) 16

**Example strategy for Web of Science** 

(critical pathways OR (clinical or critical or perioperative or surgical or integrated care or care or enhanced recovery or controlled or rapid recovery) adj3 (path or pathway*)) AND TS=(arthroplasty, replacement, hip/ or arthroplasty, replacement, knee/) all fields
(critical pathways OR (clinical or critical or perioperative or surgical or integrated care or care or enhanced recovery or controlled or rapid recovery) adj3 (path or pathway*)) AND (spin$ surg$ OR spin$ discectomy or spin$ fusion or laminectomy or laminotomy) all fields

**Legend:** Example of search strategies for academic databases used in this review.
