# Supplementary material for: Consensus‐Building Processes for Implementing Perioperative Care Pathways in Common Elective Surgeries: A Systematic Review
Source: J Adv Nurs. 2024 Oct 9;81(11):7311–31. doi: 10.1111/jan.16524 (PMC12535325; doi:10.1111/jan.16524)
Supplement: Supplementary file 3 — Data S3.: Quality appraisal of included articles using Mixed Methods Appraisal Tool (MMAT). [file JAN-81-7311-s003.docx]

Additional file 3

| **Mixed Methods Appraisal Tool** | | | | | | | | | | | | | | | | | | |
| --- | --- | --- | --- | --- | --- | --- | --- | --- | --- | --- | --- | --- | --- | --- | --- | --- | --- | --- |
|  |  |  | Quantitative randomized controlled trials | | | | | Quantitative nonrandomised | | | | | Quantitative descriptive | | | | | |
| First Author, year | S1. Are there clear research questions? | S2. Do the collected data allow to address the research questions? | 2.1. Is randomization appropriately performed? | 2.2. Are the groups comparable at baseline? | 2.3. Are there complete outcome data? | 2.4. Are outcome assessors blinded to the intervention provided? | 2.5 Did the participants adhere to the assigned intervention? | 3.1. Are the participants representative of the target population? | 3.2. Are measurements appropriate regarding both the outcome and intervention (or exposure)? | 3.3. Are there complete outcome data? | 3.4. Are the confounders accounted for high risk of n the design and analysis? | 3.5. During the study period, is the intervention administered (or exposure occurred) as intended? | 4.1. Is the sampling strategy relevant to address the research question? | 4.2. Is the sample representative of the target population? | 4.3. Are the measurements appropriate? | 4.4. Is the risk of nonresponse bias low? | 4.5. Is the statistical analysis appropriate to answer the research question? |  |
| Alvis,  2020 (1) | Yes | Yes |  |  |  |  |  | Yes | Yes | Yes | Yes | Yes |  |  |  |  |  |  |
| Angus,  2019 (2) | Yes | Yes |  |  |  |  |  | Yes | Yes | Yes | Yes | Yes |  |  |  |  |  |  |
| Barber,  2017 (3) | Yes | Yes |  |  |  |  |  | Yes | Yes | Yes | Yes | Yes |  |  |  |  |  |  |
| Bardiau,  2003 (4) | Yes | Yes |  |  |  |  |  | Yes | Yes | Yes | Yes | Yes |  |  |  |  |  |  |
| Blackburn,  2016 (5) | Yes | Yes |  |  |  |  |  |  |  |  |  |  | Yes | Yes | Can’t tell | Yes | No |  |
| Bradywood,  2017 (6) | Yes | Yes |  |  |  |  |  |  |  |  |  |  | Yes | Yes | Yes | No | Yes |  |
| Chung,  2012 (7) | Yes | Yes |  |  |  |  |  |  |  |  |  |  | Yes | Yes | Yes | Yes | Yes |  |
| Cook,  2008 (8) | Yes | Yes |  |  |  |  |  | Yes | Yes | Yes | Yes | Yes |  |  |  |  |  |  |
| Didden,  2019 (9) | Yes | Yes |  |  |  |  |  | Yes | Yes | Yes | Yes | Yes |  |  |  |  |  |  |
| Eklund,  2022 (10) | Yes | Yes |  |  |  |  |  |  |  |  |  |  | Yes | Yes | Can’t tell | Yes | Yes |  |
| El Othmani,  2021 (11) | Yes | Yes |  |  |  |  |  |  |  |  |  |  | Yes | Yes | Yes | Can’t tell | Yes |  |
| Featherall,  2018 (12) | Yes | Yes |  |  |  |  |  | Yes | Yes | Yes | Yes | Yes |  |  |  |  |  |  |
| Feng,  2019 (13) | Yes | Yes |  |  |  |  |  | Yes | Yes | Yes | Can’t tell | Yes |  |  |  |  |  |  |
| Foni,  2020 (14) | Yes | Yes |  |  |  |  |  | Yes | Yes | Yes | Yes | Yes |  |  |  |  |  |  |
| Garg,  2021 (15) | Yes | Yes |  |  |  |  |  | Yes | Yes | Yes | Can’t tell | Yes |  |  |  |  |  |  |
| Gayed,  2013 (16) | Yes | Can’t tell |  |  |  |  |  |  |  |  |  |  | Yes | Yes | Yes | Yes | Yes |  |
| Ghobrial,  2020 (17) | Yes | Yes |  |  |  |  |  | Yes | Yes | Yes | Yes | Yes |  |  |  |  |  |  |
| Gulotta,  2011 (18) | Yes | Yes |  |  |  |  |  | Yes | Yes | Yes | Yes | Yes |  |  |  |  |  |  |
| Gwynne-Jones,  2017 (19) | Yes | Yes |  |  |  |  |  | Yes | Yes | Yes | Can’t tell | Yes |  |  |  |  |  |  |
| Hall,  2019 (20) | No | Can’t tell |  |  |  |  |  |  |  |  |  |  | Yes | Yes | Yes | Yes | Can’t tell |  |
| Hawasli,  2020 (21) | Yes | Yes |  |  |  |  |  | Yes | Yes | Yes | Can’t tell | Yes |  |  |  |  |  |  |
| Hebl,  2008 (22) | Yes | Yes |  |  |  |  |  | Yes | Yes | Yes | Yes | Yes |  |  |  |  |  |  |
| Hypnar,  2001 (23) | Can’t tell | Can’t tell |  |  |  |  |  |  |  |  |  |  |  |  |  |  |  |  |
| Improta,  2015 (24) | Yes | Yes |  |  |  |  |  | Yes | No | Yes | Yes | Yes |  |  |  |  |  |  |
| Lampilas,  2021 (25) | Yes | Yes |  |  |  |  |  | Yes | Yes | Yes | Yes | No |  |  |  |  |  |  |
| Larsen,  2008 (26) | Yes | Yes |  |  |  |  |  | Yes | Yes | Yes | Yes | Yes |  |  |  |  |  |  |
| Larsen,  2008 (27) | Yes | Yes | Yes | Yes | Yes | Yes | Yes |  |  |  |  |  |  |  |  |  |  |  |
| Li,  2018 (28) | Yes | Yes |  |  |  |  |  | Yes | Yes | Yes | No | Yes |  |  |  |  |  |  |
| Lin,  2011 (29) | Yes | Yes |  |  |  |  |  | Yes | Yes | Yes | Can’t tell | Yes |  |  |  |  |  |  |
| Loftus,  2014 (30) | Yes | Yes |  |  |  |  |  | Yes | Yes | Yes | Yes | No |  |  |  |  |  |  |
| Macdonald,  2005 (31) | Can’t tell | Can’t tell |  |  |  |  |  |  |  |  |  |  |  |  |  |  |  |  |
| Mertes,  2013 (32) | Yes | Yes |  |  |  |  |  | No | Yes | Yes | Yes | Can’t tell |  |  |  |  |  |  |
| Mudumbai,  2016 (33) | Yes | Yes |  |  |  |  |  |  |  |  |  |  | Yes | Yes | Yes | Yes | Yes |  |
| Parkes,  2021 (34) | Yes | Yes | Yes | Yes | Yes | Can’t tell | yes |  |  |  |  |  |  |  |  |  |  |  |
| Pearson,  2000 (35) | Yes | Yes |  |  |  |  |  | Can’t tell | Can’t tell | Yes | No | Can’t tell |  |  |  |  |  |  |
| Pennington,  2003 (36) | Yes | Yes |  |  |  |  |  |  |  |  |  |  | Yes | Yes | Yes | Yes | Yes |  |
| Peters,  2006 (37) | Yes | Yes |  |  |  |  |  | Yes | Yes | Can’t tell | Can’t tell | Yes |  |  |  |  |  |  |
| Raphael,  2011 (38) | Yes | Yes |  |  |  |  |  | Yes | Yes | Yes | No | Yes |  |  |  |  |  |  |
| Riepen,  2021 (39) | Yes | Yes |  |  |  |  |  | Yes | Yes | Yes | Can’t tell | Yes |  |  |  |  |  |  |
| Scanlon,  2004 (40) | No | Can’t tell |  |  |  |  |  | Yes | Yes | Can’t tell | No | Yes |  |  |  |  |  |  |
| Schubert, 2021 (41) | Yes | Yes |  |  |  |  |  | Yes | Yes | Yes | Yes | Yes |  |  |  |  |  |  |
| Scott,  2013 (42) | Yes | Yes |  |  |  |  |  | Yes | Yes | Yes | Yes | Yes |  |  |  |  |  |  |
| Shao,  2022 (43) | Yes | Yes |  |  |  |  |  | Yes | Yes | Yes | Yes | Yes |  |  |  |  |  |  |
| Shaw,  2016 (44) | Yes | Yes |  |  |  |  |  | Yes | Yes | Yes | Can’t tell | Yes |  |  |  |  |  |  |
| Shields,  2017 (45) | Yes | Yes |  |  |  |  |  | Yes | Yes | Yes | Can’t tell | Yes |  |  |  |  |  |  |
| Sivaganesan,  2019 (46) | Yes | Yes |  |  |  |  |  | Yes | Yes | Yes | Yes | Yes |  |  |  |  |  |  |
| Smith,  2019 (47) | Yes | Yes |  |  |  |  |  | Yes | Yes | Yes | Can’t tell | Yes |  |  |  |  |  |  |
| Soffin,  2019 (48) | Yes | Yes |  |  |  |  |  | Yes | Yes | Yes | No | Yes |  |  |  |  |  |  |
| Soffin,  2019 (49) | Yes | Yes |  |  |  |  |  | Yes | Yes | Yes | Yes | Yes |  |  |  |  |  |  |
| Soffin,  2020 (50) | Yes | Yes |  |  |  |  |  | Yes | Yes | Yes | Yes | Yes |  |  |  |  |  |  |
| Stowers,  2016 (51) | Yes | Yes |  |  |  |  |  | Yes | Yes | Yes | No | Yes |  |  |  |  |  |  |
| Stratton,  2000 (52) | Yes | Can’t tell |  |  |  |  |  |  |  |  |  |  | Can’t tell | Can’t tell | Yes | Can’t tell | Can’t tell |  |
| Van der Sluis,  2015 (53) | Yes | Yes |  |  |  |  |  | Yes | Yes | Yes | Yes | Yes |  |  |  |  |  |  |
| Vanhaecht,  2005 (54) | Yes | Yes |  |  |  |  |  | Yes | Yes | Yes | Yes | Yes |  |  |  |  |  |  |
| Walker,  2020 (55) | Yes | Yes |  |  |  |  |  | Yes | Yes | Yes | Yes | Yes |  |  |  |  |  |  |
| Wallny,  2014 (56) | Yes | Can’t tell |  |  |  |  |  |  |  |  |  |  | Yes | Yes | Can’t tell | Can’t tell | Can’t tell |  |
| Walter,  2007 (57) | No | Yes |  |  |  |  |  |  |  |  |  |  | Yes | Can’t tell | Yes | No | Yes |  |
| Wang,  2020 (58) | Yes | Yes |  |  |  |  |  |  |  |  |  |  | Yes | Yes | Yes | Yes | Can’t tell |  |
| Wang,  2022 (59) | Yes | Yes |  |  |  |  |  | Yes | Yes | Yes | Yes | Yes |  |  |  |  |  |  |
| Wang,  2022 (60) | Yes | Yes |  |  |  |  |  | Yes | Yes | Yes | Yes | Yes |  |  |  |  |  |  |
| Woo,  2000 (61) | No | Yes |  |  |  |  |  | Yes | No | Can’t tell | Can’t tell | Can’t tell |  |  |  |  |  |  |
| Yang,  2016 (62) | Yes | Yes | Yes | Yes | Yes | Yes | Yes |  |  |  |  |  |  |  |  |  |  |  |
| Young,  2021 (63) | Yes | Yes |  |  |  |  |  | Yes | Yes | Yes | Yes | Yes |  |  |  |  |  |  |

Additional File 3: Quality appraisal of included articles using Mixed Methods Appraisal Tool (MMAT). *From:* Hong QN, Gonzalez-Reyes A, Pluye P. Improving the usefulness of a tool for appraising the quality of qualitative, quantitative and mixed methods studies, the Mixed Methods Appraisal Tool (MMAT). J Eval Clin Pract. 2018;24(3):459-467. doi:10.1111/jep.12884

**REFERENCES**

1. Alvis BD, Amsler RG, Leisy PJ, Feng X, Shotwell MS, Pandharipande PP, et al. Effects of an anesthesia perioperative surgical home for total knee and hip arthroplasty at a Veterans Affairs Hospital: a quality improvement before-and-after cohort study. Can J Anaesth. 2021;68(3):367-75.

2. Angus M, Jackson K, Smurthwaite G, Carrasco R, Mohammad S, Verma R, et al. The implementation of enhanced recovery after surgery (ERAS) in complex spinal surgery. J Spine Surg. 2019 Mar;5(1):116-123.

3. Barber C, Fraser JF, Mendez GG, Bradley B, Loftus TJ, Jacofsky DJ. The Halo Effect: An Unintended Benefit of Care Pathways. J Knee Surg. 2017;30(3):264-8.

4. Bardiau FM, Taviaux NF, Albert A, Boogaerts JG, Stadler M. An Intervention Study to Enhance Postoperative Pain Management. Anesth Analg. 2003;96(1).

5. Blackburn J, Madhavan P, Leung YL, Walburn M. An enhanced recovery program for elective spinal surgery patients. J Clin Outcomes Manag. 2016;23(10):462-9.

6. Bradywood A, Farrokhi F, Williams B, Kowalczyk M, Blackmore CC. Reduction of Inpatient Hospital Length of Stay in Lumbar Fusion Patients With Implementation of an Evidence-Based Clinical Care Pathway. Spine. 2017;42(3):169-76.

7. Chung S-B, Lee S-H, Kim E-S, Eoh W. Implementation and outcomes of a critical pathway for lumbar laminectomy or microdiscectomy. J Korean Neurosurg Soc. 2012;51(6):338-42.

8. Cook JR, Warren M, Ganley KJ, Prefontaine P, Wylie JW. A comprehensive joint replacement program for total knee arthroplasty: a descriptive study. BMC Musculoskelet Disord. 2008;9:154.

9. Didden AGM, Punt IM, Feczko PZ, Lenssen AF. Enhanced recovery in usual health care improves functional recovery after total knee arthroplasty. Int J Orthop Trauma Nurs. 2019;34:9-15.

10. Eklund SE, Vodonos A, Ryan-Barnett SM. Changing practice to increase rates of spinal anaesthesia for total joint replacement. J Perioper Pract. 2022;32(4):83-9.

11. El-Othmani MM, Crespi Z, Pallekonda V, Sayeed Z, Saleh KJ. The Implementation of Lean Six Sigma Principles to Improve the Value of Care Delivery for Total Joint Arthroplasty Patients: The Perioperative Institute of Surgical Excellence Experience. J Am Acad Orthop Surg. 2021;29(21):E1087-E96.

12. Featherall J, Brigati DP, Faour M, Messner W, Higuera CA. Implementation of a Total Hip Arthroplasty Care Pathway at a High-Volume Health System: Effect on Length of Stay, Discharge Disposition, and 90-Day Complications. J Arthroplasty. 2018;33(6):1675-80.

13. Feng C, Zhang Y, Chong F, Yang M, Liu C, Liu L, et al. Establishment and Implementation of an Enhanced Recovery After Surgery (ERAS) Pathway Tailored for Minimally Invasive Transforaminal Lumbar Interbody Fusion Surgery. World Neurosurg. 2019;129:e317-e23.

14. Foni NO, Costa LAV, Paiao ID, Oliveira IO, Carvalho RT, Lenza M, et al. Clinical pathway improves medical practice in total knee arthroplasty. PLoS One. 2020;15(5):e0232881.

15. Garg B, Mehta N, Bansal T, Shekhar S, Khanna P, Baidya DK. Design and Implementation of an Enhanced Recovery After Surgery Protocol in Elective Lumbar Spine Fusion by Posterior Approach: A Retrospective, Comparative Study. Spine. 2021;46(12).

16. Gayed B, Black S, Daggy J, Munshi IA. Redesigning a joint replacement program using Lean Six Sigma in a Veterans Affairs hospital. JAMA Surg. 2013;148(11):1050-6.

17. Ghobrial GM, Wilson J, Franco D, Vogl K, Vaccaro AR, Harrop JS. Implementation of a Spine-Centered Care Pathway at a Regional Academic Spine Center. Global Spine J. 2020;10(1 Suppl):36S-40S.

18. Gulotta LV, Padgett DE, Sculco TP, Urban M, Lyman S, Nestor BJ. Fast Track THR: One Hospital's Experience with a 2-Day Length of Stay Protocol for Total Hip Replacement. HSS J. 2011;7(3):223-8.

19. Gwynne-Jones DP, Martin G, Crane C. Enhanced Recovery After Surgery for Hip and Knee Replacements. Orthop Nurs. 2017;36(3):203-10.

20. Hall KM, Moreno JR, Dosselman L, Bagley CA. Implementation of an enhanced recovery pathway in complex spine surgery patients in a multidisciplinary center. Clinical Neurosurgery. 2019;66(Supplement 1):127.

21. Hawasli AH, Ray WZ, Goad MA, Frank TL, Ellis ER, Schmidt M, et al. Project management for developing a spine "enhanced recovery after surgery" program in a large university-affiliated hospital. J Neurosurg Sci. 2020;64(2):206-12.

22. Hebl JR, Dilger JA, Byer DE, Kopp SL, Stevens SR, Pagnano MW, et al. A pre-emptive multimodal pathway featuring peripheral nerve block improves perioperative outcomes after major orthopedic surgery. Reg Anesth Pain Med. 2008;33(6):510-7.

23. Hypnar LA, Anderson L. Attaining superior outcomes with joint replacement patients. J Nurs Adm. 2001;31(11):544-9.

24. Improta G, Balato G, Romano M, Carpentieri F, Bifulco P, Alessandro Russo M, et al. Lean Six Sigma: a new approach to the management of patients undergoing prosthetic hip replacement surgery. J Eval Clin Pract. 2015;21(4):662-72.

25. Lampilas A, Bouyer B, Ferrero E, Khalifé M, Bergeot A, Guigui P, et al. Evaluation of enhanced recovery after spine surgery: Specificities in an academic public hospital. Orthop Traumatol Surg Res. 2021;107(7):103027.

26. Larsen K, Hvass KE, Hansen TB, Thomsen PB, Søballe K. Effectiveness of accelerated perioperative care and rehabilitation intervention compared to current intervention after hip and knee arthroplasty. A before-after trial of 247 patients with a 3-month follow-up. BMC Musculoskelet Disord. 2008;9(1):59.

27. Larsen K, Sørensen OG, Hansen TB, Thomsen PB, Søballe K. Accelerated perioperative care and rehabilitation intervention for hip and knee replacement is effective: a randomized clinical trial involving 87 patients with 3 months of follow-up. Acta Orthop. 2008;79(2):149-59.

28. Li J, Li H, Xv Z-k, Wang J, Yu Q-f, Chen G, et al. Enhanced recovery care versus traditional care following laminoplasty: A retrospective case-cohort study. Medicine. 2018;97(48).

29. Lin P-C, Hung S-H, Wu H-F, Hsu H-C, Chu C-Y, Su S-J. The effects of a care map for total knee replacement patients. J Clin Nurs. 2011;20(21-22):3119-27.

30. Loftus T, Agee C, Jaffe R, Tao J, Jacofsky DJ. A simplified pathway for total knee arthroplasty improves outcomes. J Knee Surg. 2014;27(3):221-8.

31. MacDonald V, Arthur B, Parent S. The Vancouver General Hospital joint replacement Rapid Recovery Program: optimizing outcomes through focused pathways. Journal of Orthopaedic Nursing. 2005;9(2):95-102.

32. Mertes SC, Raut S, Khanduja V. Integrated care pathways in lower-limb arthroplasty: are they effective in reducing length of hospital stay? Int Orthop. 2013;37(6):1157-63.

33. Mudumbai SC, Walters TL, Howard SK, Kim TE, Lochbaum GM, Memtsoudis SG, et al. The Perioperative Surgical Home model facilitates change implementation in anesthetic technique within a clinical pathway for total knee arthroplasty. Healthcare. 2016;4(4):334-9.

34. Parkes RJ, Ayeko O, Brunton L, Griffiths-Jones W, Ungvari Z, Goss H. Revolutionising rapid recovery: a quality improvement project in hip and knee replacement. BMJ Open Qual. 2021;10(2):e001249.

35. Pearson S, Moraw I, Maddern GJ. Clinical pathway management of total knee arthroplasty: a retrospective comparative study. Aust N Z J Surg. 2000;70(5):351-4.

36. Pennington JM, Jones DP, McIntyre S. Clinical pathways in total knee arthroplasty: a New Zealand experience. J Orthop Surg (Hong Kong).2003;11(2):166-73.

37. Peters CL, Shirley B, Erickson J. The Effect of a New Multimodal Perioperative Anesthetic Regimen on Postoperative Pain, Side Effects, Rehabilitation, and Length of Hospital Stay After Total Joint Arthroplasty. J Arthroplasty. 2006;21(6 Suppl 2):132-8.

38. Raphael M, Jaeger M, van Vlymen J. Easily adoptable total joint arthroplasty program allows discharge home in two days. Can J Anaesth. 2011;58(10):902-10.

39. Riepen DW, Gelvez D, Collett GA, Nakonezny P, Estrera KA, Huo MH. Standardized Total Knee Arthroplasty Pathway Improves Outcomes in Minority Patients. Am J Manag Care. 2021;27(5):e152-e6.

40. Scanlon J, Richards B. Development of a same day laminectomy program. J Perianesth Nurs. 2004;19(2):84-8.

41. Schubert A, Patterson M, Sumrall WD, Broussard D, Dias D, Aboalfaraj A, et al. Perioperative population management for primary hip arthroplasty reduces hospital and postacute care utilization while maintaining or improving care quality. J Clin Anesth. 2021;68:110072.

42. Scott NB, McDonald D, Campbell J, Smith RD, Carey AK, Johnston IG, et al. The use of enhanced recovery after surgery (ERAS) principles in Scottish orthopaedic units--an implementation and follow-up at 1 year, 2010-2011: a report from the Musculoskeletal Audit, Scotland. Arch Orthop Trauma Surg. 2013;133(1):117-24.

43. Shao X, Li R, Zhang L, Jiang W. Enhanced Recovery After Surgery Protocol for Oblique Lumbar Interbody Fusion. Indian J Orthop. 2022;56(6):1073-82.

44. Shaw RA, Pilot P. How are fast-track programmes like rapid recovery delivering value across hospitals, cultures and healthcare systems in orthopedics within a cost-constrained environment? Value in Health. 2016;19(7):A535.

45. Shields LB, Clark L, Glassman SD, Shields CB. Decreasing hospital length of stay following lumbar fusion utilizing multidisciplinary committee meetings involving surgeons and other caretakers. Surg Neurol Int. 2017;8:5.

46. Sivaganesan A, Wick JB, Chotai S, Cherkesky C, Stephens BF, Devin CJ. Perioperative Protocol for Elective Spine Surgery Is Associated With Reduced Length of Stay and Complications. J Am Acad Orthop Surg. 2019;27(5):183-9.

47. Smith J, Probst S, Calandra C, Davis R, Sugimoto K, Nie L, et al. Enhanced recovery after surgery (ERAS) program for lumbar spine fusion. Perioper Med (Lond). 2019;8(1):4.

48. Soffin EM, Vaishnav AS, Wetmore DS, Barber L, Hill P, Gang CH, et al. Design and Implementation of an Enhanced Recovery After Surgery (ERAS) Program for Minimally Invasive Lumbar Decompression Spine Surgery: Initial Experience. Spine. 2019;44(9).

49. Soffin EM, Wetmore DS, Barber LA, Vaishnav AS, Beckman JD, Albert TJ, et al. An enhanced recovery after surgery pathway: association with rapid discharge and minimal complications after anterior cervical spine surgery. Neurosurg. 2019;46(4):E9.

50. Soffin EM, Beckman JD, Tseng A, Zhong H, Huang RC, Urban M, et al. Enhanced Recovery after Lumbar Spine Fusion. Anesthesiology. 2020;133(2):350-63.

51. Stowers MD, Manuopangai L, Hill AG, Gray JR, Coleman B, Munro JT. Enhanced Recovery After Surgery in elective hip and knee arthroplasty reduces length of hospital stay. ANZ J Surg. 2016;86(6):475-9.

52. Stratton L. Improving Systems and Care for Patients Receiving Total Joint Replacement. Disease Management. 2000;3(2):83-97.

53. van der Sluis G, Goldbohm RA, Bimmel R, Galindo Garre F, Elings J, Hoogeboom TJ, et al. What Augmented Physical Activity and Empowerment Can Bring to Patients Receiving Total Knee Replacement: Content, Implementation, and Comparative Effectiveness of a New Function-Tailored Care Pathway in a Routine Care Setting. Biomed Res Int. 2015;2015:1-8.

54. Vanhaecht K, Sermeus W, Tuerlinckx G, Witters I, Vandenneucker H, Bellemans J. Development of a clinical pathway for total knee arthroplasty and the effect on length of stay and in-hospital functional outcome. Acta Orthop Belg. 2005;71(4):439-44.

55. Walker CT, Gullotti DM, Prendergast V, Radosevich J, Grimm D, Cole TS, et al. Implementation of a Standardized Multimodal Postoperative Analgesia Protocol Improves Pain Control, Reduces Opioid Consumption, and Shortens Length of Hospital Stay After Posterior Lumbar Spinal Fusion. Neurosurgery. 2020;87(1):130-6.

56. Wallny TA, Strauss A, Goldmann G, Oldenburg J, Wirtz DC, Pennekamp PH. Elective total knee arthroplasty in haemophilic patients Proposal for a clinical pathway. Hamostaseologie. 2014;34(4A):S23-S9.

57. Walter FL, Bass N, Bock G, Markel DC. Success of clinical pathways for total joint arthroplasty in a community hospital. Clin Orthop. 2007;457:133-7.

58. Wang P, Wang Q, Kong C, Teng Z, Li Z, Zhang S, et al. Enhanced recovery after surgery (ERAS) program for elderly patients with short-level lumbar fusion. J Orthop Surg Res. 2020;15(1):299.

59. Wang W, Wang P, Kong C, Teng Z, Zhang S, Sun W, et al. Retrospective Data Analysis for Enhanced Recovery After Surgery (ERAS) Protocol for Elderly Patients with Long-Level Lumbar Fusion. World Neurosurg. 2022;164:e397-e403.

60. Wang S, Wang P, Li X, Sun W, Kong C, Lu S. Enhanced recovery after surgery pathway: association with lower incidence of wound complications and severe hypoalbuminemia in patients undergoing posterior lumbar fusion surgery. World Neurosurg. 2022;164:e397-e403.

61. Woo T, Bramwell M, Greenwood B, Gow S, Ackerman-Rainville R, Corradetti P, et al. Integrated systems to reduce length of stay for knee and hip joint replacement surgeries. Healthc Manage Forum. 2000;13(3):60-2.

62. Yang GMD, Chen WMD, Chen WMD, Tang XMD, Huang YMD, Zhang LMD. Feasibility and Safety of 2-Day Discharge After Fast-Track Total Hip Arthroplasty: A Chinese Experience. J Arthroplasty. 2016;31(8):1686-92.e1.

63. Young R, Cottrill E, Pennington Z, Ehresman J, Ahmed AK, Kim T, et al. Experience with an Enhanced Recovery After Spine Surgery protocol at an academic community hospital. Journal of Neurosurgery: Spine. 2021;34(4):680-7.
