# Supplementary material for: Consensus‐Building Processes for Implementing Perioperative Care Pathways in Common Elective Surgeries: A Systematic Review
Source: J Adv Nurs. 2024 Oct 9;81(11):7311–31. doi: 10.1111/jan.16524 (PMC12535325; doi:10.1111/jan.16524)
Supplement: Supplementary file 4 — Data S4.: Count of studies that utilised single method or category only. [file JAN-81-7311-s002.docx]

**Additional file 4**

**Table S1**: Count of studies that utilised single method or category only

| **Category or method in consensus process** | **Frequency (%) of studies**  **N = 62** |
| --- | --- |
| ***Disciplines involved in consensus process*** |  |
| Clinical | 17 (27%) |
| Non-clinical | 1 (2%) |
| Leadership | 2 (3%) |
| Clinical, non-clinical | 12 (19%) |
| Clinical, leadership | 7 (11%) |
| Non-clinical, leadership | 1 (2%) |
| Clinical, non-clinical, leadership | 14 (23%) |
| ***How items were defined*** |  |
| Information gathering and/or processing | 23 (37%) |
| Clinician determined | 2 (3%) |
| Information gathering and/or processing, clinician determined | 25 (40%) |
| Information gathering and/or processing, externally defined | 2 (3%) |
| Information gathering and/or processing, clinician determined, externally defined | 4 (6%) |
| ***Methods of operationalising pathways*** |  |
| Implementation strategy focussed | 37 (60%) |
| Theory or framework | 0 (0%) |
| Implementation strategy focussed, theory or framework | 10 (16%) |
| ***Evaluate pathways*** |  |
| Clinical outcomes* | 37 (60%) |
| Compliance outcomes | 2 (3%) |
| Clinical and compliance outcomes | 22 (35%) |

* Clinical outcomes referred to include measures of length of stay, mortality rates, post-operative complications and post-operative hospital readmission rates.
